# RETRACTED: Meligy et al. Therapeutic Potential of Mesenchymal Stem Cells Versus Omega n − 3 Polyunsaturated Fatty Acids on Gentamicin-Induced Cardiac Degeneration. *Pharmaceutics* 2022, *14*, 1322

**DOI:** 10.3390/pharmaceutics18070801

**Published:** 2026-06-29

**Authors:** Fatma Y. Meligy, Hanan Sharaf El-Deen Mohammed, Tarek M. Mostafa, Mohamed M. Elfiky, Israa El-Sayed Mohamed Ashry, Ahmed M. Abd-Eldayem, Nermin I. Rizk, Dina Sabry, Eman S. H. Abd Allah, Salwa Fares Ahmed

**Affiliations:** 1 Histology and Cell Biology Department, Faculty of Medicine, Assiut University, Assiut 71526, Egypt; 2 Internal Medicine_Critical Care Department, Faculty of Medicine, Assiut University, Assiut 71526, Egypt; 3 Anatomy and Embryology Department, Faculty of Medicine, Assiut University, Assiut 71526, Egypt; 4 Anatomy and Embryology Department, Faculty of Medicine, Menoufia University, Menoufia 32952, Egypt; 5 Pharmacology Department, Faculty of Medicine, Assiut University, Assiut 71526, Egypt; 6 Clinical Physiology Department, Faculty of Medicine, Menoufia University, Menoufia 32952, Egypt; 7 Medical Biochemistry and Molecular Biology Department, Faculty of Medicine, Badr University in Cairo, Cairo 11829, Egypt; 8 Medical Biochemistry and Molecular Biology Department, Faculty of Medicine, Cairo University, Cairo 11562, Egypt; 9 Medical Physiology Department, Faculty of Medicine, Assiut University, Assiut 71526, Egypt

The journal retracts the article titled “Therapeutic Potential of Mesenchymal Stem Cells versus Omega n − 3 Polyunsaturated Fatty Acids on Gentamicin-Induced Cardiac Degeneration” [1], cited above.

Following the publication of the manuscript, concerns were brought to the attention of the Editorial Office regarding the integrity of several figures published in this article [1].

In accordance with the journal’s standard procedures, the Editorial Office and Editorial Board conducted an investigation that confirmed inappropriate editing had taken place within Figures 1, 6 and 9. Although the authors cooperated during the investigation, the explanations and supporting materials provided were not deemed sufficient by the Editorial Board to resolve the identified integrity issues and establish the provenance of the images.

Consequently, the Editorial Board has lost confidence in the reliability of the findings and decided to retract this publication [1], as per MDPI’s retraction policy.

This retraction was approved by the Editor-in-Chief of the journal *Pharmaceutics.*

The authors have been informed of this retraction and have indicated their disagreement with this decision.
